# Clinicopathological Characteristics and Survival Outcomes of Gastrointestinal Neuroendocrine Tumors in a Large Safety Net Hospital

**DOI:** 10.3390/jcm15051811

**Published:** 2026-02-27

**Authors:** Ramya Singhal, Grace H. Kim, Isa Jacoba, Qing Zhao, Haesook T. Kim, Horst C. Weber

**Affiliations:** 1 Boston Medical Center, Boston, MA 02118, USA; 2 Chobanian & Avedisian School of Medicine, Boston University, Boston, MA 02118, USA; 3 Dana-Farber Cancer Institute, Boston, MA 02215, USA; 4 VA Boston Healthcare System, Boston, MA 02132, USA

**Keywords:** neuroendocrine tumor (NET), gastrointestinal (GI) tract, overall survival, survival risks, safety net hospital, racial differences

## Abstract

**Background/Objectives**: Neuroendocrine tumors (NET) are heterogeneous, rare tumors predominantly of the gastrointestinal (GI) tract. Overall survival (OS) and prognostic factors of GI-NETs remain poorly understood. This study aimed to determine the clinicopathological characteristics and OS outcomes of GI-NETs in a demographically diverse population. **Methods**: All patients at a large tertiary safety-net hospital with a pathology-proven GI-NET diagnosis from 2001 through 2022 were identified. Demographic and tumor characteristics were collected from medical charts. For survival analysis, the Kaplan-Meier method and Cox regression analysis were used for group comparison. **Results**: A total of 222 cases were identified: 208 at six primary GI sites and 14 liver metastases only). Of these primary sites, rectum was the most frequent (27.4%). OS was the highest for appendiceal NETs (5-year OS: 95%) and lowest for stomach NETs (5-year OS 76%). Prognostic factors included age (5-year OS: 92% vs. 67% for <65 and ≥65, respectively, *p* < 0.0001), stage (5-year OS: 89%, 91%, 84% and 50% for stage I, II, III, IV, respectively, *p* = 0.01), size (5-year OS: 91%, 91% and 75% for <1 cm, 1–2 cm, >2 cm, respectively, *p* = 0.0025), and grade (5-year OS: 92% and 39% for well/moderately and poorly differentiated, respectively, *p* < 0.0001). In multivariable analysis, age (hazard ratio (HR) 3.33, *p* = 0.0002), high tumor stage (HR 2.24, *p* = 0.02), larger tumor size (HR 2.76, *p* = 0.0028), and poor grade (HR 6.03, *p* = 0.0003) were significantly associated with poor OS whereas race and educational level were not significantly associated with OS. **Conclusions**: In this large, single-site retrospective analysis of GI-NET, we report the anatomical distribution of GI NETs and survival among GI-NETs. Survival among various GI-NETs is generally favorable. These novel findings expand on our knowledge of GI-NET survival and prognostication.

## 1. Introduction

Neuroendocrine tumors (NETs) are a group of heterogeneous, rare tumors caused by the malignant transformation of neuroendocrine cells. They can arise in several anatomical sites throughout the body, with the most common sites of origin being the lungs and the gastrointestinal (GI) tract. Approximately 60% of all NETs occur in the GI tract, with the small intestine reported as the most frequent site of origin, followed by the rectum, colon and stomach [1,2,3,4]. Tissues of GI-NETs are positive for one or more of the neuroendocrine tumor differentiation markers including synaptophysin, chromogranin A, or neuron specific enolase (NSE) [5]. While most of these tumors are asymptomatic, some have the capability to secrete biologically active molecules causing distinct clinically overt hormonal access syndromes, such as carcinoid syndrome [4,6], with approximately 40% of GI-NETs considered to be functional [3,4,7].

The incidence rate of NETs overall has increased more than 6-fold worldwide over the last few decades likely related to increased screening and diagnostic capabilities, but also due to increased recognition and recent changes to the classification system of NETs throughout the years [2,3,8]. Previously labeled as “carcinoid” tumors that are slow-growing and often non-malignant, recent classification systems highlight the malignant potential of these NETs and largely rely on their immunohistochemical sensitivity to aid diagnosis and grading [2,9,10,11,12,13].

Risk factors significantly associated with development of GI-NETs include genetic predisposition, proton pump inhibitor use, age over 60 [14], and high-fat diet [15].

Although the incidence of GI-NETs has been increasing worldwide [9], prognostic factors associated with survival outcomes have not been well studied. Most prior studies on risk factors and demographic profiles of NETs have used data from the National Cancer Institute’s Surveillance, Epidemiology, and End Results (SEER) program with significantly limited data acquisition on patient outcomes, tumors localized to GI sites, and racial/ethnic factors [2,9,10,16,17,18]. A recent analysis of SEER GI NET data demonstrated that non-Hispanic Black patients had better cause-specific survival in comparison to non-Hispanic White patients, suggesting that race/ethnicity is an independent prognostic factor in patients with GI-NETs [19]. This study, however, had significant limitations in generalizability as it describes a population with a low proportion of ethnic/racial minorities and lacked patient-level socioeconomic information. Additionally, there was no information on tumor grade, a recognized prognostic indicator. Lastly, a GI-NET-specific comparison of survival outcomes is very limited outside the SEER- and National Cancer Database-based reports with the known methodological limitations [2,9,10,16,20].

Owing to the significant knowledge gap of tumor characteristics, survival outcomes, and associated risk factors in GI-NETs based on anatomic site, we conducted a single-site retrospective study of clinicopathological characteristics and survival outcomes of GI-NETs in a racially and socioeconomically diverse tertiary safety net hospital.

## 2. Materials and Methods

### 2.1. Study Design & Population

A cross-sectional, population-based retrospective study was conducted at Boston Medical Center (BMC), an urban academic medical center that serves as the largest safety-net hospital in New England. This study was approved by the BMC Institutional Review Board and was in accordance with the ethical standards of the institutional and national research committee and with the 1995 Helsinki Declaration. Due to the nature of this retrospective, population-based study and the preserved anonymity of patients, a waiver of informed consent was approved by the BMC IRB.

Patients at BMC with a pathology-proven GI-NET diagnosis between 2001 and 2022 were identified via the CoPath database of the Department of Pathology which facilitated the direct query of all patients at the hospital with a surgical resection specimens or biopsy-proven tissue diagnosis of NETs. In addition, the Clinical Data Warehouse (CDW) for Research was used to identify patients with NET diagnoses based on ICD-9 (209.x) and ICD-10 (D3A.x; C7A.x; C7B.x; Z85.060) codes and documented keywords for NET biomarkers chromogranin A and synaptophysin in the electronic health records (EHR). A total of 222 unique participants were identified with a NET diagnosis between 1 January 2001 and 31 December 2022. A manual chart review of each patient’s pathology specimen results was then conducted to ensure samples were accurately identified as NETs originating in the GI tract.

### 2.2. Data Collection

Patient and tumor characteristics, including age at diagnosis, race, sex, educational level, tumor site, stage, grade, size, NET markers (synaptophysin, chromogranin), and date of death (if applicable) were collected from the EHR of the identified patients. Utilizing TNM information, samples were classified into stage I-IV.

Given that the study period spans for more than 20 years of case reviews, there are significant limitations in GI-NET classification for tumor grading as there have been three iterations of classification updates during this time period (2000, 2010, and 2019) using different classification criteria [21,22,23]. While currently the 2019 WHO GI-NET classification is used in conjunction with minor updates in 2022 [12], this classification for tumor grading was the first to unify the approach for GI and pancreatic neuroendocrine neoplasms. In our GI-NET cohort, the availability of tumor grade by degree of differentiation was most complete, in contrast to other criteria. We therefore opted to present tumor grade according to the degree of differentiation. A detailed description of tumor grade data procurement and handling of missing data is provided in the Supplementary Material. Manual chart review was performed to corroborate and input any missing data after initial data extraction by the CDW.

### 2.3. Statistical Analysis

Patient and tumor characteristics were reported descriptively and compared using Fisher’s exact test, χ^2^ test or Wilcoxon rank-sum test, as appropriate. The primary endpoint was overall survival (OS), defined as time from the date of histologically proven diagnosis to death from any cause. Patients who were alive at the time of last contact were censored. OS was estimated using the Kaplan-Meier method, and the log-rank test was used for group comparison. Multivariable regression analysis for OS was performed using Cox proportional hazards model. Factors that were considered in multivariable model included GI site, age, sex, race, ethnicity, educational level, tumor size, tumor stage and tumor grade. Prior to modelling, linearity and proportional hazards assumptions were examined. Age was dichotomized using the methods of restricted cubic spline function on log relative hazard and classification and regression tree for survival data [24,25,26]. A category for missing data was included in the multivariable model. Ki-67 was not considered due to insufficient data. All *p*-values were two-sided at a significance level of 0.05. All calculations were performed using SAS 9.4 (SAS Institute, Inc., Cary, NC, USA) and R version 4.3.1.

## 3. Results

### 3.1. Patients

A total of 208 patients with primary GI-NETs at six different anatomical sites and 14 patients with metastatic NET disease to the liver as the only detectable tumor manifestation (without known primary site) were identified. A comprehensive list of demographic and tumor characteristics is summarized in Table 1. Of the total 208 patients with primary GI-NETs, 101 patients are female (48.6%) and 107 (51.4%) are male. Median age at diagnosis was 55 years (range: 17, 86). 73 patients identified as White (35.1%), while 86 patients identified as Black (41.3%) and 10 patients as Asian (4.8%). With regards to educational level, about two thirds of all patients had High school or College degrees (Table 1). The majority of patients (63%) were diagnosed with stage I or II disease whereas only about 20% were at stage III and IV. Staging information was not available for 32 patients (15.4%). Close to half of all GI-NET patients were diagnosed with tumors under 1 cm as the greatest dimension measured, whereas about one quarter (24.5%) presented with tumors larger than 2 cm. Notably, because the study spanned over 20 years, we assessed differences in patient demographics between 2002–2011 and 2012–2022 and found that demographic characteristics were similar across the two periods except for organ sites (Supplementary Table S1). Compared to the 2002–2011 period, more stomach and fewer rectum and small intestine NETs were identified during 2012–2022.

Baseline demographic characteristics for patients with liver metastatic NETs of unknown primary site were similar to those with primary GI-NETs except these patients were significantly older (median age 55 vs. 68, *p* = 0.007). Characteristics of liver metastases were significantly different with a more advanced tumor stage, larger tumor size, and higher tumor grade when compared with primary GI-NETs (Table 1).

### 3.2. Tumor Characteristics

Of all primary GI-NETs (N = 208), most localized to the rectum (N = 57, 27.4%), followed by pancreas (N = 47, 22.6%), small bowel (N = 41, 19.7%), stomach (N = 28, 13.5%), appendix (N = 22, 10.6%), and colon (N = 13, 6.3%) (Table 2). The distribution of GI sites was significantly different between White and Black patients (*p* = 0.007). A higher incidence of rectum, and small bowel NETs was identified in Black patients, in comparison to higher incidence of pancreatic NETs in White patients (Table 3).

### 3.3. Survival Outcomes

**Figure 1:**
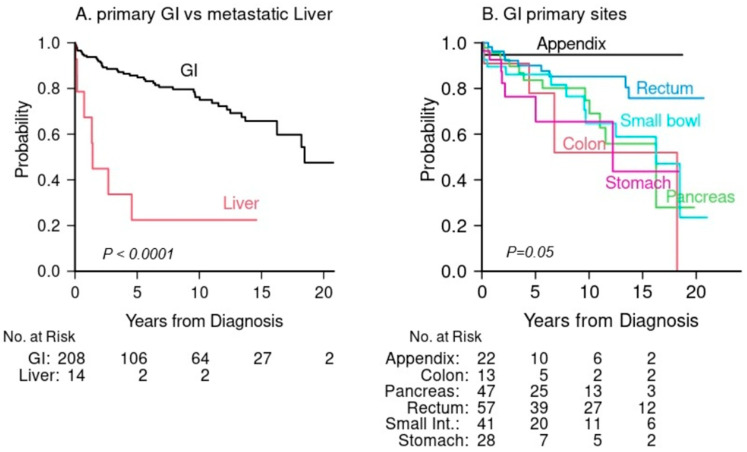
Overall survival for primary GI-NETs vs. liver metastases (**A**) and for anatomical GI-NET sites (**B**).

**Figure 2:**
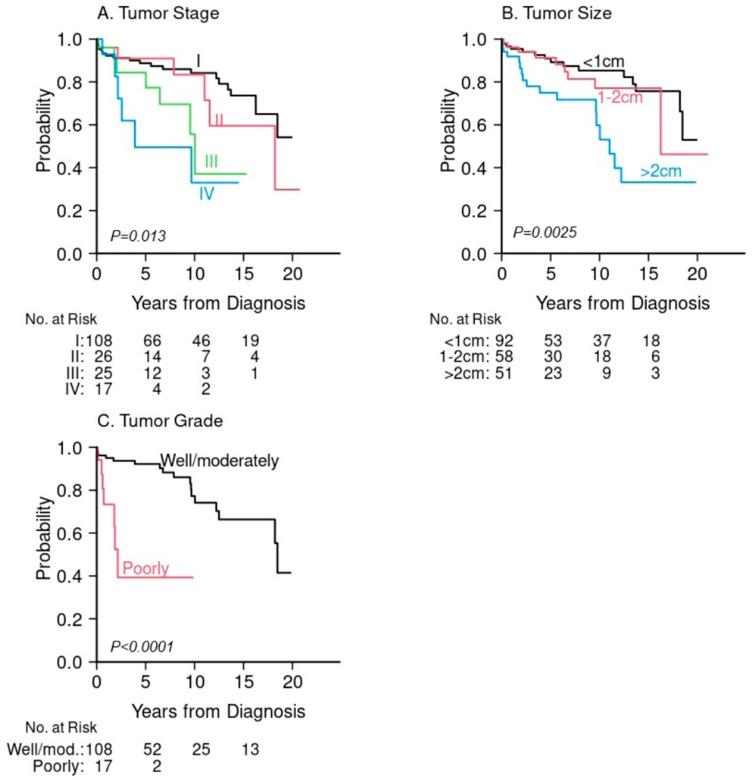
Overall survival for primary GI-NETs only. (**A**) Tumor Stage. (**B**) Tumor Size. (**C**) Tumor Grade.

**Figure 3:**
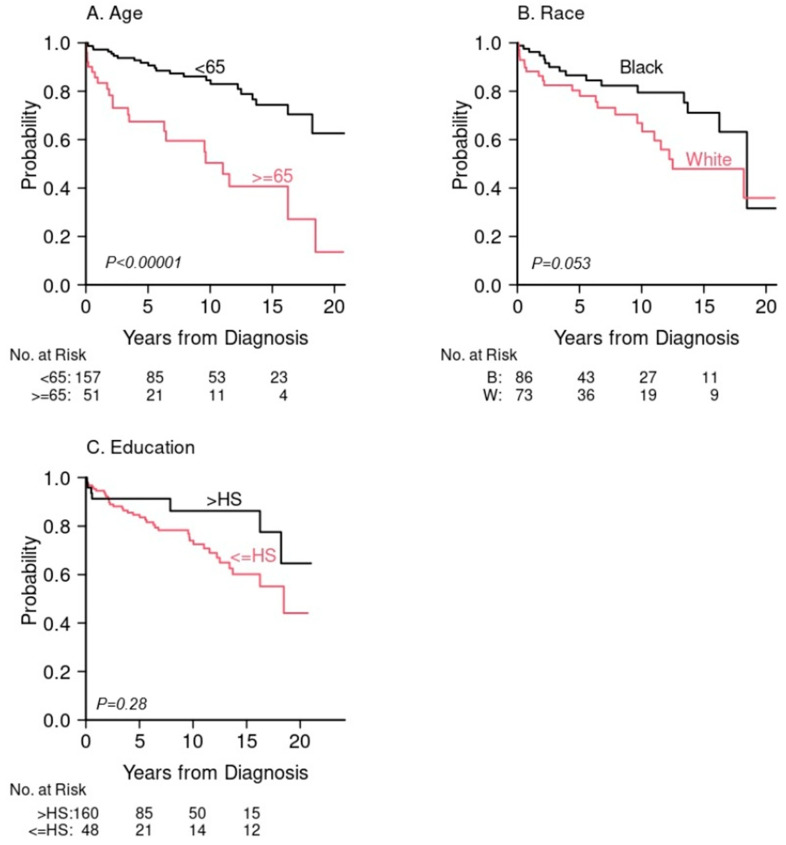
Overall survival for primary GI-NETs only. (**A**) Age. (**B**) Race. (**C**) Education.

For the entire cohort, the median follow-up time among survivors was 5.9 years (range 0.2, 21). Overall survival at 5 years was 86% (95% confidence interval (CI): 79%, 90%) for primary GI-NETs and 22% (95% CI: 3.5%, 51%) for liver metastases (Figure 1A). For the primary GI-NETs cohort, survival outcomes varied by primary site. Patients with appendiceal NETs demonstrated the highest 5-year OS (95%), while those with stomach NETs had the lowest (76%). (Figure 1B, Table 3). Tumor stage (5-year OS: 89%, 91%, 84% and 50% for tumor stage I, II, III, IV, respectively, *p* = 0.01) was significantly associated with poor overall survival, as well as tumor size (5-year OS: 91%, 91%, 75% for size < 1 cm, 1–2 cm, >2 cm, respectively, *p* = 0.0025), and tumor grade (5-year OS: 92% for well/moderately-differentiated vs. 39% for poorly differentiated, *p* < 0.0001) (Figure 2A–C, Table 3). Similarly, when OS was stratified by G tumor grades G1, G2, and G3 when available, OS was significantly shortened for high grade GI-NETs (5-year OS: 94%, 83% and 45% for G1, G2, G3, respectively, *p* = 0.009. Supplementary Figure S1). Among demographic factors, older age was significantly associated with poor OS (5-year OS: 67% for age ≥ 65 compared to 92% age < 65, *p* < 0.0001) (Table 3, Figure 3A). Black patients had somewhat better survival compared to White patients, but this difference was not statistically significant (5-year OS: 87% vs. 80%, respectively, *p* = 0.053) (Table 3, Figure 3B). Higher educational level (college or higher) was not statistically significantly associated with OS compared to high school education of lower (5-year OS: 91% vs. 86%, *p* = 0.28) (Table 3, Figure 3B,C). We further compared overall survival between Black and White patients for each anatomical GI-NET site and no site was significantly different between these two groups due largely to the small sample size in each site (Supplementary Figure S2). Because the incidence of organ sites were slightly different between 2002–2011 and 2012–2022 (Supplementary Table S1), we compared survival outcome and found no difference between these two periods. The 5-year OS was 87% (95% CI: 80%, 92%) in 2002–2011 and 82% (95% CI: 69%, 90%) in 2012–2022 (*p* = 0.69).

In multivariable Cox regression analysis for overall survival, consistent with the results from the univariable analysis, advanced tumor stage (hazard ratio (HR) 2.24 for III/IV vs. I/II, *p* = 0.02), large tumor size (HR 2.76 for >2 cm vs. ≤2 cm, *p* = 0.0028) and poorly differentiated grade (HR 6.03, *p* = 0.0003) were associated with an increased risk of death (Table 3). Regarding tumor site, tumors located in the colon (HR 4.65, *p* = 0.015), pancreas/small bowel (HR 2.36, *p* = 0.026) and stomach (HR 4.79, *p* = 0.0029) were associated with a higher risk of death compared to appendix/rectum. Furthermore, old age was significantly associated with an increased risk of death (HR 3.33 for age ≥ 65 vs. <65, *p* = 0.0002). Race, however, was not associated with an increased risk of death (HR 1.49 for White vs. Black patients, *p* = 0.16) (Table 3).

### 3.4. Symptoms

The presence or absence of symptoms prompting diagnosis was reported for 217 patients of the total cohort. Of these, 78 (35.1%) patients were asymptomatic at time of diagnosis. Within this subset, diagnosis was made incidentally on imaging (33.3%), screening colonoscopy (57.7%) or surveillance endoscopy (8.9%). For those patients with symptoms at time of diagnosis, abdominal pain was the most common presenting symptom (N = 65, 83%). None of the GI-NETs were determined to be hormonally active or resulted in clinical manifestations of carcinoid syndrome.

### 3.5. Treatment Modalities

Specification of treatment modality utilized was reported for 221 patients of the total cohort (Supplementary Table S2). Within this subset, therapeutic removal of GI-NET was executed in conjunction with diagnosis in majority of the rectal and appendiceal cases, prompted by polyp removal during screening or surveillance endoscopy and urgent appendectomy, respectively. Chemotherapy was the predominant form of treatment utilized in metastatic cases, in accordance with standard of care practices. Peptide receptor radiation therapy was not used in any of these study participants. While FDG-PET/CT does not have a proven routine role in the evaluation or management of GI-NETs (in contrast to thorax and neck) according to guidelines [22], it was utilized in 32 cases (14%) of the entire cohort.

## 4. Discussion

Owing to the multitude of tissues of origin and the scarcity of NET cases overall, there is a significant knowledge gap regarding anatomic site-specific tumor biology and survival outcomes. This has resulted in important limitations of clinical trial design and clinical management regarding NETs.

National data registries such as the SEER or National Cancer Database demonstrated significant increases of NET incidence rates nationally over the past 4 decades, though global incidence rates and site distribution of GI-NETs in Europe and Asia may differ from those in North America [9]. Overall, the scarcity of these studies limit accurate representation of survival outcomes and tumor characteristics, especially with possible underrepresentation of patients from lower socioeconomic status [17,18,27]. Therefore, our large long-term retrospective study of GI-NET carries a particular strength of reporting detailed clinicopathological data and survival outcomes regarding 6 anatomical sites of GI-NETs in an ethnically and socioeconomically diverse population.

Our study’s primary GI-NET population is predominantly male, Black/African American (41%, Table 1), with a high school educational level or lower (for those reported; >60%) and age in the mid 50’s at time of diagnosis. This overall demographic profile is distinct from that reported in the SEER 18 registry database, which comprises of a predominantly female, White-identifying population with age above 60 at diagnosis [28]. The differences noted in overall survival and tumor site distribution in our study compared to national database reviews could point to the environmental and biologic effects on NET oncogenesis in different population cohorts and warrants further examination.

The SEER registry data from 2000–2018 demonstrated highest prevalence of primary GI-NETs in the small intestine (21.6%), followed by rectum (16.4%) and pancreas (16.4%) [29]. Distinct from anatomical GI-NET distribution shown in SEER data, our findings show a larger portion of rectal NETs which could be due to the over-representation of Black patients compared to the SEER data, but substantially fewer colon NETs than in reports of the 2019 National Cancer Database and a recent retrospective observational study [16,20]. It is a reasonable hypothesis that the demographic makeup of our sample population, comprised of a high proportion of Black patients (>40%), may have affected the GI-NET anatomical distribution. White and Black patients together accounted for a large majority in this GI-NET study sample.

In our GI-NET sample, prevalence rates were highest for rectum, small intestine NETs in Black patients, which were significantly different from the higher prevalence of pancreatic NETs in White patients (*p* = 0.007). The high prevalence of rectal NETs in Black patients is consistent with prior literature, although the subtype distribution between Black and White patients is uniquely different from prior studies [19,30,31]. Prior SEER database studies showed the incidence rate of rectal NETs was found to be three-to six-fold higher in Black patients than White patients [32].

The reason for higher proportion of rectal NETs in Black, Hispanic, and Asian patients with known global variation remains uncertain and was speculated to be associated with healthcare access and literacy [33]. Although the rising utility of endoscopic surveillance over the years due to technological advances could partially explain increased detection of rectal NETs, one could speculate the role of socioeconomic barriers in the healthcare experiences of patients that could lead to increased detection of rectal tumors in Black patients secondary to unwarranted time delay before diagnosis, and maybe associated with yet unidentified confounding factors.

In this study, we determined overall survival of all 6 different anatomical locations of GI-NETs. Patients with appendiceal and rectal GI-NETs had the most favorable OS (5-year OS: 95%, 90%, respectively) whereas patients with stomach and colon GI NETs (5-year OS: 76%, 78%, respectively) had the poorest OS. Overall, the difference in OS across all six sites was borderline significant (*p* = 0.053) due largely to the small cases of a few sites. These findings are similar to a recent report from the SEER database [16]. Risk factors for poor survival identified in our study include age, tumor stage, size, and grade similar to previous database studies [2,9,10,16,20]. Older age has been demonstrated to worsen outcomes in prior studies [34], likely in part due to increased comorbid conditions, less aggressive treatment, and frailty. For liver metastatic lesions without a known primary site, OS was significantly lower when compared with all GI-NETs which is also congruent with aforementioned database studies and a study of pancreatic NETs [35].

In contrast to prior reports [2,9,10,16,20], our study uniquely demonstrated a similar OS in Black patients compared to their White counterparts with GI-NETs in the setting of a single site large safety net hospital study; this was noted across all GI-NET subtypes. This is distinctly different from prior studies, which have demonstrated possible association between non-Hispanic black patients with poor survival in stomach, small intestine and pancreatic NETs. Furthermore, although rectal NETs are less aggressive and exhibit more favorable prognoses, other studies have found that they exhibit higher mortality in Black or male patients than White or female patients [16,36] whereas no racial differences were detected in this regard in this study. There have been investigations showing a significant influence of social determinants of health on cancer incidence and mortality in general, with Black patients historically shown to have more aggressive tumor biology and worse outcomes [37], making our findings increasingly intriguing. Possible explanations of lack of OS differences in Black versus White patients in our study might include an association with improved healthcare delivery in the safety net hospital environment.

While our study contributes novel data to our knowledge of NETs, there are several limitations, many of which are inherent to being a retrospective study. First, data collection for such a long study period of 21 years was challenging to result in a complete data set despite diligent attention to detail. Because of missing data, certain data analyses could not be performed on factors that could affect prognosis, including comorbid conditions, treatment modalities, functionality of tumor, and family history. We were not able to identify any patients with MEN-1 syndrome, carcinoid, and hormone access syndromes [4]. Although this is surprising given literature notes the prevalence of functional NETs to be approximately 10–30% [4], we suspect this difference might be due to obscure mild cases of functional hormone access states, the inability to recognize hormone access states, or perhaps documentation failures in the beginning of electronic medical records era. Further, modifications of the GI-NET tumor grade classification changed during the study period several times and reporting had to be harmonized [21]. Although one major strength of this study sample was the racial distribution with a large group of Black patients, the small number of Asian and Hispanic patients precluded data analyses of these two groups for OS and risk factors.

## 5. Conclusions

NETs are very rare tumors overall, with the majority arising in the GI tract. Because of their rarity and multifocal localization even within the GI tract, their biology and survival risks remain poorly studied. Consequently, important limitations of clinical trial design and management regarding NETs exist despite the increasing GI-NET incidence rates.

This long-term retrospective study from a large safety net hospital setting provided novel insights into demographic and tumor-related characteristics and OS of GI-NETs emanating from six different anatomical sites, which uncovered differences and similarities compared to previously reported data. NET-related biological characteristics such as larger tumor size and higher stage and grade were associated with poor OS, as was older age. In contrast to previous large database studies, this study did not show a significant association of GI-NET OS with race and educational level.

These intriguing results warrant further examination of the tumor site specific biology of GI-NETs, risk factors for survival, and whether specific demographic factors in the setting of safety net healthcare delivery might affect outcomes.

## Figures and Tables

**Table 1:** Demographic Characteristics of Patients with Gastrointestinal Neuroendocrine Tumors (GI-NET).

	GI (Primary)		Liver (Metastasis)		*p*-Value
	N	%	N	%	
Total	208	100	14	100	
Age at Diagnosis					0.007
median (range)	55 (17, 86)		68 (41, 86)		
Sex					1
Female	101	48.6	7	50	
Male	107	51.4	7	50	
RACE					0.92
White	73	35.1	6	42.9	
Black/African American	86	41.3	4	28.6	
Asian	10	4.8	1	7.1	
Other	21	10.1	2	14.3	
Declined/Not Available	18	8.7	1	7.1	
ETHNICITY					0.87
Not Hispanic or Latino	161	77.4	10	71.4	
Hispanic or Latino	36	17.3	3	21.4	
Declined/Not Available	11	5.3	1	7.1	
Educational Level					0.06
No schooling	12	5.8			
<8th grade	14	6.7	4	28.6	
High School/GED	104	50	5	35.7	
College or higher	48	23.1	3	21.4	
Not reported	30	14.4	2	14.3	
Tumor Stage					<0.001
I	108	51.9			
II	26	12.5			
III	25	12			
IV	17	8.2	14	100	
Unavailable	32	15.4			
Tumor Size					<0.001
<1 cm	92	44.2			
1–2 cm	58	27.9	1	7.1	
>2 cm	51	24.5	8	57.1	
Unavailable	7	3.4	5	35.7	
Tumor Grade					0.007 *
Well differentiated	104	50	3	21.4	
Moderately differentiated	4	1.9	1	7.1	
Poorly differentiated	17	8.2	4	28.6	
Unavailable	83	39.9	6	42.9	

*: unavailable was not counted in the *p*-value calculation.

**Table 2:** Anatomical Distribution of Gastrointestinal Neuroendocrine Tumor (GI-NET) sites.

		All		Black		White	
	Organ	N	%	N	%	N	%
GI (primary)	Appendix	22	10.6	4	4.7	13	17.8
	Colon	13	6.3	6	7.0	3	4.1
	Pancreas	47	22.6	13	15.1	22	30.1
	Rectum	57	27.4	29	33.7	13	17.8
	Small intestine	41	19.7	21	24.4	15	20.5
	Stomach	28	13.5	13	15.1	7	9.6
	Total	208	100	86	100	73	100
Liver (metastasis)	Liver	14	100				

*p*-value for difference in site distribution between Black and White is 0.007.

**Table 3:** Univariable and multivariable analysis of overall survival (OS) for patients with Gastrointestinal Neuroendocrine Tumors (GI-NETs) only.

Univariable Analysis		N	5-yr OS (95% CI)	*p*-Value *
Age	<65	157	92% (85, 76)	<0.0001
	≥65	51	67% (51, 79)	
Race	Black	86	87% (76, 93)	0.053
	White	73	80% (68, 88)	
Educational Level	High School/GED or lower	130	86% (78, 92)	0.28
	College or higher	48	91% (78, 97)	
Organ	Appendix	22	95% (68, 99)	0.051
	Colon	13	78% (35, 94)	
	Pancreas	47	84% (67, 92)	
	Rectum	57	90% (78, 96)	
	Small intestine	41	86% (69, 94)	
	Stomach	28	76% (51, 90)	
Tumor Stage	I	108	89% (81, 94)	0.01
	II	26	91% (68, 98)	
	III	25	84% (58, 95)	
	IV	17	50% (17, 76)	
Tumor Size	<1 cm	92	91% (82, 96)	0.0025
	1–2 cm	58	91% (78, 97)	
	>2 cm	51	75% (58, 86)	
Tumor grade	Well/moderately differentiated	108	92% (84, 96)	<0.0001
	Poorly differentiated	17	39% (11, 67)	
**Multivariable analysis**			HR (95% CI)	*p*-value
Age	≥65 vs. <65		3.33 (1.77, 6.28)	0.0002
Race	White vs. Black		1.6 (0.83, 3.08)	0.16
	Other vs. Black		0.57 (0.22, 1.48)	0.25
Education level	≤HS vs. >HS		1.87 (0.76, 4.59)	0.17
GI organ	Colon vs. Appendix/Rectum		4.65 (1.35, 16)	0.015
	Pancreas/Small Bowel vs. Appendix/Rectum		2.36 (1.11, 5.04)	0.026
	Stomach vs. Appendix/Rectum		4.79 (1.71, 13.4)	0.0029
Tumor stage	III/IV vs. I/II		2.24 (1.12, 4.47)	0.02
Tumor size	>2 cm vs. ≤ 2 cm		2.76 (1.42, 5.35)	0.0028
Tumor grade	Poorly vs. well/moderately differentiated		6.03 (2.3, 15.8)	0.0003

*: log-rank test. HS: high school. The survival analyses were determined for primary GI-NETs only.

## Data Availability

Deidentified individual participant data that underlie the reported results will be made available 12 months after publication for a period of 1 year after the publication date to researchers who provide a sound proposal. The data will be provided in compliance with general applicable privacy laws, BMC and BMC IRB specific policies, data protection, and requirements for consent and anonymization. Proposals for access should be sent to christian.weber@bmc.org.

## Supplementary Materials

The following supporting information can be downloaded at: https://www.mdpi.com/article/10.3390/jcm15051811/s1. Supplementary Material and Methods. Supplementary Figure S1. Overall survival according to tumor G grade for primary GI-NETs only. Supplementary Figure S2. Comparison of Overall Survival Between Black and White Participants with Primary GI-NETs and Liver Metastases. Supplementary Table S1. Baseline Characteristic of Patients with Gastrointestinal Neuroendocrine Tumors According to Years of Diagnosis. Supplementary Table S2. Reported Treatment Modalities in Study Participants with Primary GI-NETs and Liver Metastases.

## Abbreviations

The following abbreviations are used in this manuscript:

GI-NET	Gastrointestinal Neuroendocrine Tumor
NET	Neuroendocrine Tumor
OS	Overall Survival
MEN-I	Multiple Endocrine Neoplasia Type I
SEER	Surveillance, Epidemiology, and End Results
EHR	Electronic Health Records
