# Supplementary material for: Clinicopathological Characteristics and Survival Outcomes of Gastrointestinal Neuroendocrine Tumors in a Large Safety Net Hospital
Source: J Clin Med. 2026 Feb 27;15(5):1811. doi: 10.3390/jcm15051811 (PMC12986369; doi:10.3390/jcm15051811)
Supplement: Supplementary file 1 [file jcm-15-01811-s001.zip › Suppl Mat and Methods-tracked.pdf]

# Supplementary Materials

## Materials and Methods

### Data Collection

Among the 222 patients (208 primary GI-NETs and 14 liver metastases), 106 had data on degree of tumor differentiation, and 84 had tumor G grade data. We first examined the concordance between tumor G grade and degree of differentiation. Of the 59 patients with both tumor grade and differentiation data available, 57 showed concordant classifications (concordance rate: 97%). Specifically, among 49 patients with G1/G2 tumors, 47 were classified as well differentiated, 1 G2 tumor was classified as moderately differentiated, and 1 G2 tumor was classified as poorly differentiated. All 10 patients with G3 tumors were classified as poorly differentiated. In addition, survival curves stratified by G tumor grade (Supplementary Figure 2) closely resembled those stratified by degree of differentiation (Figure 2C). Based on these findings, we used G tumor grade to assign differentiation status for the 27 patients who had G tumor grade data but missing differentiation data. Using this now validated approach, we achieved the most robust data set used for survival analyses by tumor grade as determined by degree of differentiation.
