# Supplementary material for: Clinicopathological Characteristics and Survival Outcomes of Gastrointestinal Neuroendocrine Tumors in a Large Safety Net Hospital
Source: J Clin Med. 2026 Feb 27;15(5):1811. doi: 10.3390/jcm15051811 (PMC12986369; doi:10.3390/jcm15051811)
Supplement: Supplementary file 1 [file jcm-15-01811-s001.zip › Supplementary Figure S1-tracked.pdf]

Supplementary Figures

Supplementary Figure S1. Overall survival according to tumor G grade for primary GI-NETs only.

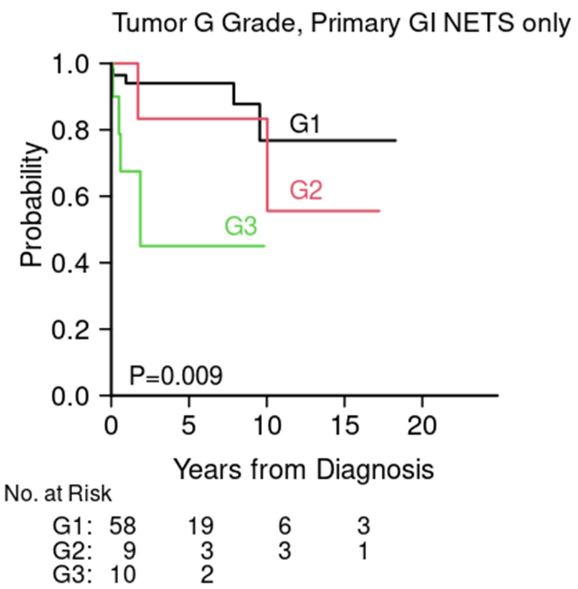

Tumor G Grade	N	5-yr OS (95% CI)	p-value
G1	59	94% (82, 98)	0.009
G2	9	83% (27, 97)	
G3	10	45% (8, 78)	

Overall survival (OS) and 5-year OS of primary GI-NETs stratified by tumor grades G1, G2, and G3 differs significantly (P=0.009) and resembles that of OS stratified by degree of tumor differentiation.
