# Supplementary material for: Clinicopathological Characteristics and Survival Outcomes of Gastrointestinal Neuroendocrine Tumors in a Large Safety Net Hospital
Source: J Clin Med. 2026 Feb 27;15(5):1811. doi: 10.3390/jcm15051811 (PMC12986369; doi:10.3390/jcm15051811)
Supplement: Supplementary file 1 [file jcm-15-01811-s001.zip › Supplementary Figure S2-tracked.pdf]

Supplementary Figures

Supplementary Figure S2: Comparison of overall survival between Black and White participants with primary GI-NETs and liver metastases.

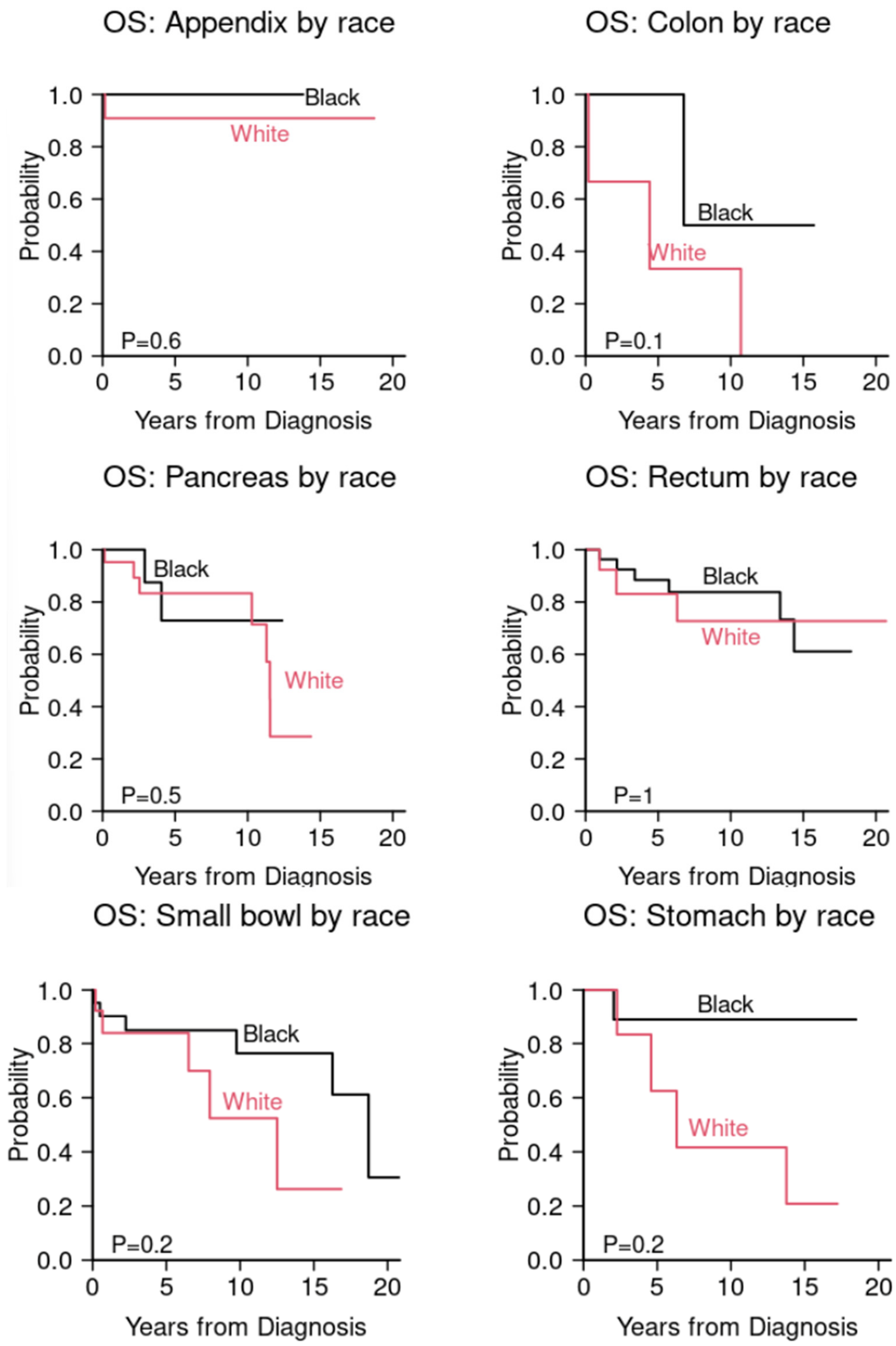

GI-NET is Gastrointestinal Neuroendocrine Tumors; OS is Overall Survival
