# Supplementary material for: Clinicopathological Characteristics and Survival Outcomes of Gastrointestinal Neuroendocrine Tumors in a Large Safety Net Hospital
Source: J Clin Med. 2026 Feb 27;15(5):1811. doi: 10.3390/jcm15051811 (PMC12986369; doi:10.3390/jcm15051811)
Supplement: Supplementary file 1 [file jcm-15-01811-s001.zip › Supplementary Table S1-tracked.pdf]

Supplementary Table

Supplementary Table S1. Baseline characteristic of patients with gastrointestinal neuroendocrine tumors according to years of diagnosis

	2002-2011		2012-2022		P-value
	N	%	N	%	
Total	129	100	79	100	
Age at Diagnosis					0.53
median (range)	54 (17, 86)		56 (17, 81)		
Sex					1
Female	63	48.8	38	48.1	
Male	66	51.2	41	51.9	
RACE					0.49
White	45	37.8	28	39.4	
Black / African American	51	42.9	35	49.3	
Asian	8	6.7	2	2.8	
Other	15	12.6	6	8.5	
ETHNICITY					0.85
Not Hispanic or Latino	97	82.2	64	81	
Hispanic or Latino	21	17.8	15	19	
Educational Level					0.48
No schooling	9	8.2	3	4.4	
<8th grade	8	7.3	6	8.8	
High School/GED	67	60.9	37	54.4	
College or higher	26	23.6	22	32.4	
Tumor Stage					0.1
I	73	65.8	35	53.8	
II	11	9.9	15	23.1	
III	15	13.5	10	15.4	
IV	12	10.8	5	7.7	
Tumor Size					0.83
<1cm	56	44.8	36	47.4	
1-2 cm	38	30.4	20	26.3	
>2 cm	31	24.8	20	26.3	
Degree of differentiation					0.3
Well/moderately differentiated	54	90	54	83.1	
Poorly differentiated	6	10	11	16.9	
Organ					0.03
Appendix	11	8.5	11	13.9	
Colon	8	6.2	5	6.3	
Pancreas	31	24	16	20.3	

Rectum	40	31	17	21.5	
Small intestine	29	22.5	12	15.2	
Stomach	10	7.8	18	22.8	

Note: for the group comparison of distributions, unavailable data were excluded from the p-value calculations.
