# Supplementary material for: Clinicopathological Characteristics and Survival Outcomes of Gastrointestinal Neuroendocrine Tumors in a Large Safety Net Hospital
Source: J Clin Med. 2026 Feb 27;15(5):1811. doi: 10.3390/jcm15051811 (PMC12986369; doi:10.3390/jcm15051811)
Supplement: Supplementary file 1 [file jcm-15-01811-s001.zip › Supplementary Table S2-tracked.pdf]

Supplementary Table

<u>Supplementary Table S2:</u> Reported treatment modalities in study participants with primary GI-NETs and liver metastases

**Treatment Modality, N (%)***	**GI-NET Primaries**						**Liver Metastases**
	**Appendix**	**Colon**	**Pancreas**	**Rectum**	**Small Bowel**	**Stomach**	
Surgery	22 (100%)	7 (53.8%)	29 (61.7%)	5 (8.8%)	26 (61.9%)	15 (53.6%)	2 (14.3%)
Endoscopy	0 (0%)	6 (46.2%)	0 (0%)	46 (80.7%)	7 (16.7%)	5 (17.9%)	0 (0%)
Chemotherapy	0 (0%)	1 (7.7%)	8 (17.0%)	8 (14.0%)	2 (4.8%)	9 (32.1%)	6 (42.6%)
SSA	0 (0%)	0 (0%)	3 (6.4%)	0 (0%)	2 (4.8%)	0 (0%)	4 (28.6%)
Other	0 (0%)	0 (0%)	2 (4.3%)	0 (0%)	0 (0%)	4 (14.3%)	1 (7.1%)

*Total number of participants with indicated treatment reported. Percent reported for each anatomical site may exceed 100% due to combination therapy with 2 or more treatment modalities.

SSA is somatostatin analogue (octreotide, lanreotide); GI-NET is gastrointestinal neuroendocrine tumor.
